# Walking on the Edge: Brain Connectivity Changes in Response to Virtual Height Challenges

**DOI:** 10.1111/ejn.70131

**Published:** 2025-05-01

**Authors:** Layla Cupertino, Emanuele Los Angeles, Nathalia Mendes Pellegrino, Thayna Magalhães‐Novaes, Brenda Luciano de Souza, Mohamed Bouri, Daniel Boari Coelho

**Affiliations:** ^1^ Center for Mathematics, Computation and Cognition Federal University of ABC São Bernardo do Campo Brazil; ^2^ Biomedical Engineering Federal University of ABC São Bernardo do Campo SP Brazil; ^3^ École Polytechnique Fédérale de Lausanne Lausanne Switzerland

**Keywords:** electroencephalography, functional connectivity, gait, locomotion, neural networks, virtual reality

## Abstract

Virtual reality (VR) environments simulating height offer a unique platform to investigate neural adaptations to emotionally salient contexts during locomotion. These simulations allow for controlled analysis of motor‐cognitive interactions under perceived threat. This secondary analysis of a previously dataset aimed to explore regional and global brain network adaptations, focusing on connectivity, modularity, and centrality, during gait under neutral and height‐induced negative conditions. Seventy‐five healthy participants performed a VR task involving a virtual plank at two heights: street level (neutral) and 80 floors up (negative). EEG was recorded using 32 scalp electrodes. Functional connectivity was analyzed using local efficiency, modularity, and eigenvector centrality across frontal, central, parietal, temporal, and occipital regions during two tasks: preparation (elevator) and active walking (plank). Repeated‐measures ANOVAs examined the effects of task and condition. Frontal connectivity was significantly higher in the negative condition across tasks, suggesting increased cognitive‐emotional regulation. Central connectivity showed a task × condition interaction, with elevated values during walking under threat, indicating increased sensorimotor integration. Occipital connectivity was higher during preparation, independent of condition, likely reflecting greater visual scene processing. Modularity was reduced in the negative condition, consistent with decreased functional segregation, while eigenvector centrality was greater in frontal and parietal regions during walking, highlighting their role as integrative network hubs. Height‐related threat in VR modulates both regional and global brain network properties, enhancing integration in cognitive, motor, and visual systems. These findings advance our understanding of adaptive brain responses and support the use of VR in rehabilitation.

## Graphical abstract

Overview of EEG connectivity findings during virtual reality (VR) height exposure. (A) Participants wore a 32‐channel EEG cap while performing a VR task simulating height, consisting of two conditions (neutral vs. negative) and two tasks (preparation in the elevator vs. execution on the plank). (B) EEG connectivity was analyzed using graph‐theoretical measures: local efficiency, modularity, and eigenvector centrality. (C) Key findings included increased frontal connectivity during the negative condition across tasks, enhanced central connectivity during walking under threat, and elevated occipital connectivity during the preparation phase. Additionally, the negative condition elicited reduced modularity (suggesting decreased segregation), and walking increased eigenvector centrality in frontal and parietal regions. *Take‐home message*: VR height exposure modulates cortical network organization, promoting greater integration of cognitive, motor, and visual systems.

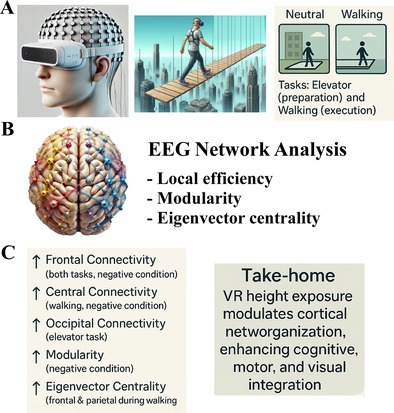

**Abbreviations**
AMICA: Adaptive mixture independent component analysis
ANOVA: Analysis of variance
EEG: Electroencephalography
HMD: Head‐mounted display
ICA: Independent component analysis
ICC: Intra‐class correlation coefficient
IPQ: Igroup Presence Questionnaire
SSQ: Simulator Sickness Questionnaire
STAI: State–Trait Anxiety Inventory
VR: Virtual reality

## 1 Introduction

Virtual reality (VR) has emerged as a transformative tool in neuroscience and psychology research, enabling the creation of highly immersive environments to simulate real‐world scenarios with unparalleled control. Among these applications, VR environments simulating height have proven particularly valuable for studying anxiety, fear, and their effects on physiological and behavioral responses. By inducing a safe but realistic perception of height, VR platforms allow researchers to investigate motor and cognitive adaptations to stress in ways that would be impractical or unsafe in real‐world settings (Cleworth et al. 2012; El Basbasse et al. 2023; Zhu et al. 2023). These simulations are widely used to explore mechanisms underlying the fear of heights (acrophobia) and examine changes in posture, gait, and mental effort under threatening conditions (Schniepp et al. 2014; Diemer et al. 2016; Peterson et al. 2018). Furthermore, VR simulations provide ecological validity in experimental paradigms, bridging the gap between controlled laboratory settings and real‐life experiences (El Basbasse et al. 2023; Raffegeau et al. 2024). This dual advantage of experimental control and realistic exposure has positioned VR height simulations as an essential tool for fundamental research and clinical applications, such as exposure therapy for individuals with visual height intolerance (Krijn et al. 2004; Schniepp et al. 2014; Wuehr et al. 2019; Zhu et al. 2024).

Neuroimaging studies, particularly those leveraging electroencephalography (EEG), have significantly advanced our understanding of how the brain processes fear and stress in VR environments simulating height. El Basbasse et al. (2023) demonstrated that immersive VR tasks, such as walking on a virtual plank suspended at a great height, effectively elicited fear, as evidenced by increased right‐hemispheric lateralization in frontal alpha activity. Similarly, Teng et al. (2024) revealed that individuals with varying levels of acrophobia exhibit distinct EEG microstate characteristics during VR height exposure, suggesting that fear intensity modulates the neural dynamics of emotional and cognitive processing. Notably, Zhu et al. (2024) examined the P300 event‐related potential in a naturalistic VR height exposure paradigm, finding stress‐induced alterations in cognitive processing, which varied across individuals depending on their stress resilience. These findings underscore the potential of VR to elucidate neural mechanisms underlying fear and stress, particularly when combined with high‐resolution EEG techniques that capture the brain's dynamic responses to environmental threats (El Basbasse et al. 2023; Zhu et al. 2023). However, while these studies have identified key neural markers of stress and fear, relatively few have explored how these processes influence connectivity within brain networks during complex motor tasks like walking.

Despite the growing body of research, critical gaps remain in understanding the neural mechanisms underlying gait adaptations in response to perceived threats in VR environments. Most prior studies have focused on static tasks or generalized neural activation patterns, leaving the intricate dynamics of brain connectivity during gait underexplored. Locomotion, particularly in anxiety‐inducing settings such as VR‐simulated heights, requires integrating motor, sensory, and cognitive processes to ensure stability and safety (Meehan et al. 2002; Schniepp et al. 2014; Raffegeau et al. 2024). Investigating these connectivity patterns can provide deeper insights into how the brain reallocates resources and adapts its networks to maintain motor control while managing heightened emotional and cognitive demands. In this context, our study aims to investigate how cerebral connectivity adapts during gait in a VR environment simulating height.

Given the complex interplay between emotion, cognition, and motor control during locomotion in threatening environments, understanding the neural mechanisms that support adaptive responses remains a critical goal. Locomotion under perceived threat requires the brain to coordinate motor, sensory, and executive processes while concurrently regulating fear and anxiety (El Basbasse et al. 2023; Zhu et al. 2023, 2024). Previous studies have shown that VR‐induced stress can modulate brain activity, with increased engagement of prefrontal regions involved in cognitive control and emotional regulation (Gage et al. 2003; Starcke and Brand 2012; Teng et al. 2024; Zhu et al. 2024). Additionally, heightened motor demands in threatening environments are often reflected in increased activity within the motor and sensorimotor networks to maintain stability and prevent falls (Schniepp et al. 2014). However, most of these studies have primarily focused on regional activations or changes in global signal amplitude, providing limited insight into the dynamic interactions across distributed brain regions that underpin adaptive responses to emotionally threatening locomotor challenges. Traditional activation‐based analyses may overlook critical aspects of functional organization, such as integrating and segregating neural networks, which are essential for coordinating simultaneous cognitive, emotional, and motor demands (Bullmore and Sporns 2009; Bassett and Sporns 2017). Moreover, while increased activity in frontal and motor areas has been documented under conditions of perceived threat, less is known about how these regions communicate as part of a coherent network to support behavioral adaptation. To address this gap, we reanalyzed an existing dataset using a network neuroscience approach, focusing on metrics such as functional connectivity, modularity, and centrality to characterize large‐scale cortical reorganization. This framework enables a more nuanced understanding of how the brain dynamically adjusts its network architecture under dual‐task demands. We hypothesize that VR‐induced height exposure will lead to increased functional connectivity within frontal networks, reflecting greater recruitment of emotional and cognitive regulatory systems and motor and sensorimotor networks, consistent with the elevated control demands for balance and locomotion in an anxiety‐provoking environment.

## 2 Methods

### 2.1 Participants

The study utilized a dataset (El Basbasse et al. 2023) involving 75 healthy participants (45 women, age 24.00 ± 3.61 years) to examine the impact of height‐induced anxiety on motor performance. Inclusion criteria required participants to be within the age range of 18 to 35 years, without any diagnosis of mental or neurodevelopmental disorders, neurological diseases (e.g., migraine, epilepsy), or extreme fear of heights. Exclusion criteria were applied in cases of mental disorders, as indicated by scores on the Beck Depression Inventory (Beck 1961) above the cutoff value (e.g., one participant with a score of 51, where values greater than or equal to 30 indicates severe depression) and for participants reporting bipolar disorder, panic disorder, or depression with addiction. Additionally, high scores on the Acrophobia Questionnaire (Baker et al. 1973) also led to exclusion, affecting one potential participant. The dataset analyzed in this study originates from a previously published experiment that the ethics committee of the Faculty of Psychology at Ruhr‐University Bochum approved. All participants provided written informed consent prior to participation, and the study was conducted following the Declaration of Helsinki.

### 2.2 EEG Recordings and Processing

EEG recordings were acquired using a mobile 32‐channel system (LiveAmp 32, Brain Products GmbH, Germany), sampled at 1000 Hz. The Fpz electrode was used as the ground electrode, while FCz was the reference electrode. The wireless amplifier used three acceleration sensors to measure head and body movements, and VR‐compatible EEG caps were adjusted using Velcro straps.

To examine the impact of height‐induced anxiety on motor performance, participants walked across a virtual plank at either 80 floors up (negative condition) or street level (neutral condition). They were placed in a virtual room representing a city environment, beginning inside an elevator located at ground level. Participants were instructed to step outside the elevator, orient themselves to the surroundings, and re‐enter the elevator. They pressed a red button using their right‐hand controller, prompting the elevator to ascend. In the neutral control condition, the elevator moved upwards only briefly before returning to street level. In the negative condition, the elevator continued to the top of the skyscraper. Upon reaching the designated height, the elevator doors opened and participants were instructed to look around for 1 min without moving or speaking—this marked the beginning of the first EEG recording segment (“elevator”). Next, they stepped onto a virtual plank and remained there for another 1‐min interval while observing the environment (“start of plank”). Finally, they walked to the end of the plank, where a third 1‐min recording took place (“end of plank”). EEG recordings were thus segmented into three standardized 60‐s epochs per condition, allowing for direct comparison between the neutral and negative conditions. Although the total duration of the task differed slightly due to the elevator ascent in the negative condition, only the three time‐locked segments were analyzed. Prior to entering VR, participants walked briefly on a real, slightly unstable wooden plank to reduce novelty effects. They were introduced to two affective ratings—subjective fear and presence in VR—which were collected after each of the three segments. The virtual reality headset (HMD) was carefully fitted over the EEG cap, and the order of neutral and negative conditions was counterbalanced across participants to control for order effects.

EEG data were preprocessed using MATLAB (Mathworks, United States) and EEGLAB 2024 (http://www.sccn.ucsd.edu/eeglab). The signal was bandpass filtered between 0.5 and 40 Hz using a zero‐phase finite impulse response (FIR) filter (pop_eegfiltnew). The signal was re‐referenced to the average reference (pop_reref). Line noise artifacts were further reduced using the CleanLine algorithm with a bandwidth of 2 Hz around 50 Hz. Automated artifact rejection was performed with the Clean Raw Data plugin, applying the following parameters: FlatlineCriterion = 5 s, ChannelCriterion = 0.8, LineNoiseCriterion = 4, BurstCriterion = 20, and WindowCriterion = 0.25. Independent component analysis was performed using the adaptive mixture independent component analysis (AMICA) algorithm (Stergiadis et al. 2022). Removed channels were interpolated using spherical splines. These steps ensured the removal of artifacts and the preservation of high‐quality data for further analysis.

The local efficiency of functional brain networks was calculated to assess the overall connectivity of EEG data. First, a connectivity matrix was constructed by estimating pairwise relationships between EEG channels using Pearson correlation. This matrix was transformed into a distance matrix, where distances were inversely proportional to the connectivity strengths. The local efficiency was computed as the mean of the inverse shortest path lengths between all pairs of nodes, quantifying the capacity for information transfer across the network. Local efficiency values range from 0 to 1, with lower values indicating inefficient, weakly connected networks and higher values reflecting efficient, strongly connected networks. This measure provides a robust quantification of functional connectivity patterns, offering insights into the integrative properties of brain networks.

To facilitate regional analysis of EEG signals, the electrodes were divided into cortical regions as follows: frontal region: FP1, FP2, Fz, F3, F7, F4, F8, FT9, FT10; temporal region: T8, T7, TP9, TP10; central region: Cz, C3, C4, FC1, FC2, FC5, FC6, CP1, CP5, CP2, CP6; parietal region: Pz, P3, P7, P4, P8; and occipital region: Oz, O1, O2. This regional grouping allowed us to analyze activity patterns in specific cortical areas, aligning with known functional divisions of the brain and enabling targeted analyses of frontal, temporal, central, parietal, and occipital activity.

To assess the functional segregation of brain networks, we computed the modularity of EEG‐derived functional connectivity matrices using the Louvain community detection algorithm. Preprocessed EEG signals were segmented into epochs corresponding to each experimental condition. Functional connectivity was estimated for each epoch using the weighted Phase Lag Index in the frequency band (1–40 Hz), resulting in symmetric adjacency matrices. These matrices were thresholded to retain the top 75% of strongest connections and binarized prior to community detection. Modularity was computed for each matrix, reflecting how the network could be partitioned into non‐overlapping modules with dense within‐module and sparse between‐module connections. Individual modularity scores were averaged across epochs for each condition. Higher values of Modularity indicate stronger modular organization, reflecting greater functional segregation between brain networks. In contrast, lower values suggest increased network integration, which may support enhanced communication across distributed regions during cognitively or emotionally demanding tasks.

Eigenvector centrality was computed to quantify the global influence of each EEG channel within the functional connectivity network. For each participant and condition, a connectivity matrix was constructed using pairwise measures of functional interaction between channels (e.g., coherence or phase‐locking values). The adjacency matrices were treated as weighted, undirected graphs, where nodes represent EEG channels and edges reflect connection strengths. Eigenvector centrality was then calculated by solving the eigenvalue problem associated with the adjacency matrix, assigning higher centrality values to nodes connected to other highly central nodes. This metric captures both direct and indirect connectivity, emphasizing the role of each channel in the overall network structure. Centrality values were extracted for each condition and electrode, and subsequently used in the statistical analysis to evaluate group and condition effects.

All analysis scripts used in this study are provided as Supporting Information.

### 2.3 Statistical Analysis

The assumption of normality was checked by using a Kolmogorov–Smirnov test.

For each variable, we performed a two‐way ANOVA, 2 (condition: neutral × negative) × 2 (task: elevator × plank walking), with repeated measures on both factors. Post hoc was performed using the Bonferroni test. Spearman's rank‐order correlation analyses were performed to explore the relationship between neural connectivity and individual differences in behavioral and subjective measures. Correlations were computed between EEG connectivity measures (e.g., frontal, central, parietal regions across tasks, and conditions) and scores from psychological questionnaires, including state and trait anxiety (STAI), avoidance and approach scales, presence and embodiment (IPQ), and simulator sickness (SSQ). The statistical analysis was conducted in STATISTICA; the significance level was set to 0.05.

## 3 Results

The normality assumption was tested using the Kolmogorov–Smirnov test for all dependent variables to assess the suitability of parametric analyses. Global efficiency data did not deviate from normality (*p* = 0.915) or modularity data (*p* = 0.915). For eigenvector centrality, most regional values followed a normal distribution: frontal (*p* = 0.217), central (*p* = 0.274), and parietal (*p* = 0.519).

**FIGURE 1:**
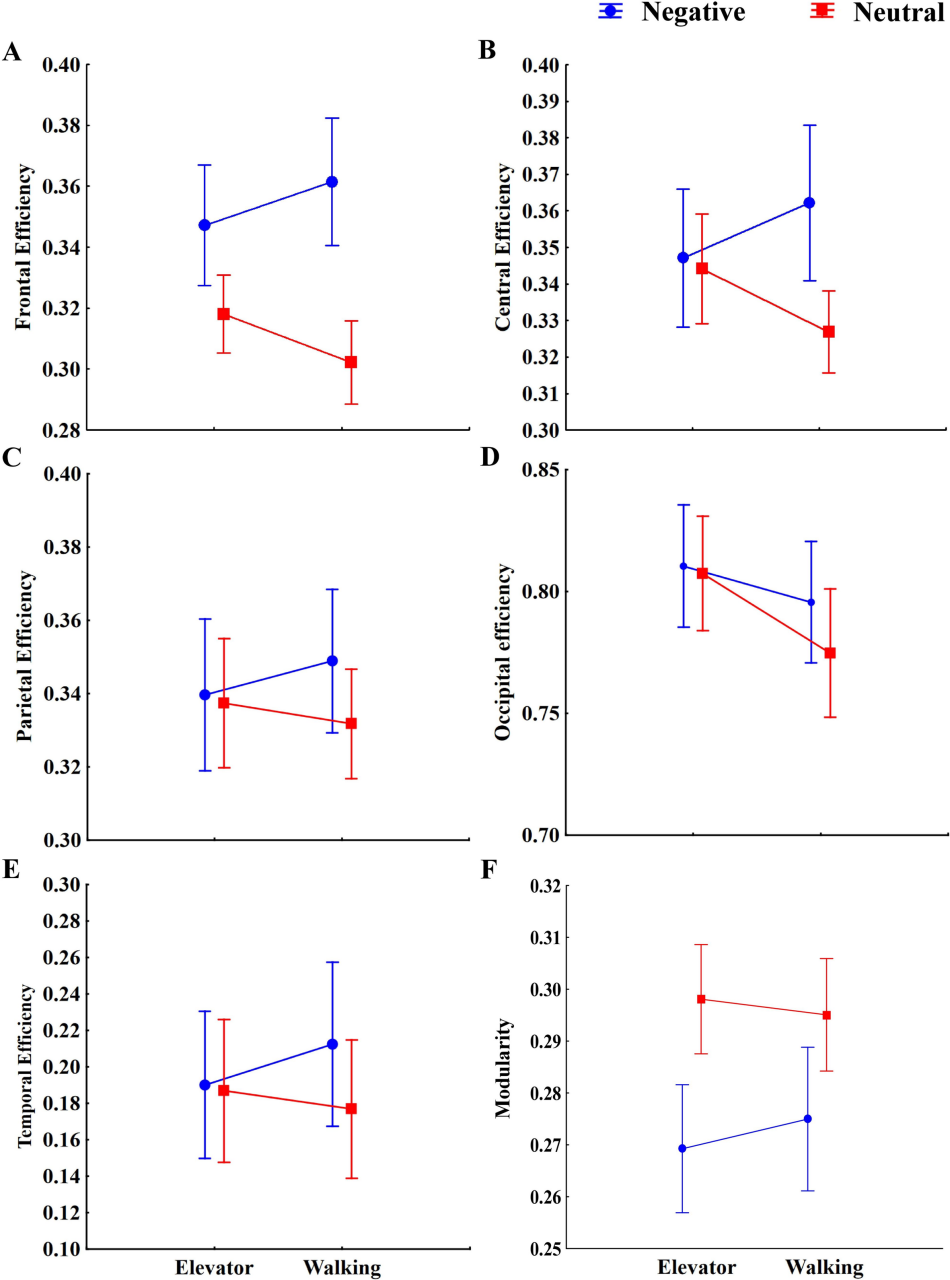
Mean and standard deviation of local connectivity of the frontal (A), central (B), parietal (C), occipital (D), and temporal (E) regions and modularity (F) for the negative and neutral conditions of the elevator and walking tasks.

For the frontal region, there was a significant difference in the condition factor (F_1,74_ = 25.01, *p* = 0.001, η^2^ = 0.25), with higher values for the negative condition (0.35 ± 0.09) than the neutral condition (0.31 ± 0.06) (Figure 1A).

For the central region, there was a significant condition × task interaction (F_1,74_ = 5.61, *p* = 0.020, η^2^ = 0.07). Post hoc comparisons using the Bonferroni correction revealed that during the walking task, connectivity was significantly higher in the negative condition (0.36 ± 0.09) compared to the neutral condition (0.33 ± 0.05; *p* = 0.001) (Figure 1B).

For the occipital region, there was a significant difference in the task factor (F_1,74_ = 9.31, *p* = 0.003, η^2^ = 0.11), with higher values for the elevator task (0.81 ± 0.11) than the walking task (0.78 ± 0.11) (Figure 1D).

No significant difference existed for the parietal (Figure 1C) and temporal (Figure 1E) regions.

For the modularity, there was a significant difference in the condition factor (F_1,74_ = 16.071, *p* = 0.001, η^2^ = 0.18), with higher values for the neutral condition (0.30 ± 0.05) than the negative condition (0.27 ± 0.06) (Figure 1F).

**FIGURE 2:**
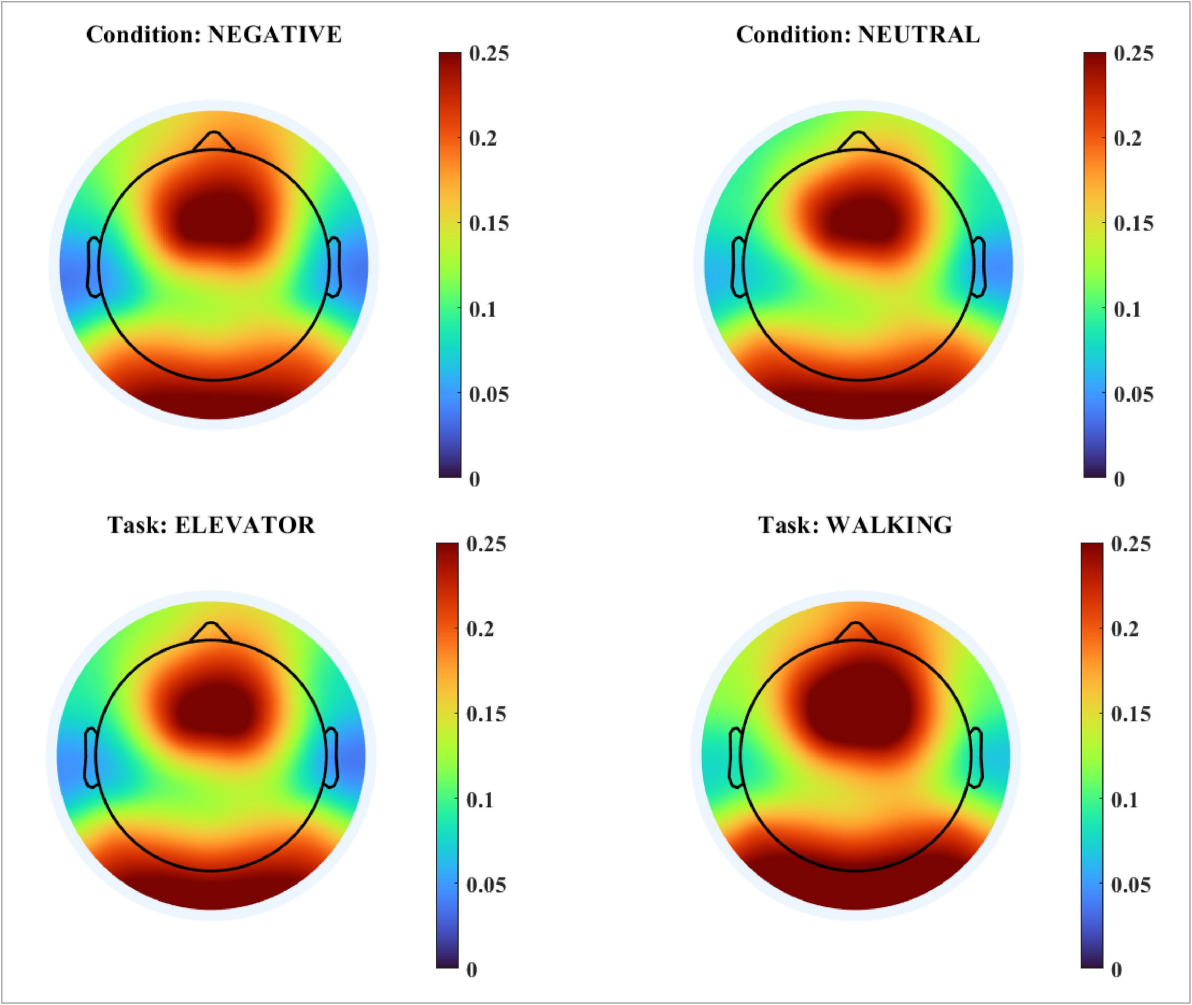
Topographic maps of eigenvector centrality averaged across all participants. The upper panels illustrate cortical centrality during the negative and neutral conditions, while the lower panels depict the elevator and walking tasks. Warmer colors indicate higher centrality values, reflecting increased global influence of the corresponding scalp regions within the functional network. Centrality was computed from EEG connectivity measures and reflects the relative importance of each region in the network topology. All maps present data using the same color scale (range 0–0.25).

The results of eigenvector centrality (Figure 2) showed a significant main effect of task in the frontal region (F₁,₇₄ = 4.41, *p* = 0.039, η^2^ = 0.06, with higher centrality during the walking task—0.20 ± 0.02 vs. 0.15 ± 0.02), and in the parietal region (F₁,₇₄ = 4.89, *p* = 0.030, η^2^ = 0.06, with higher centrality during the walking task—0.18 ± 0.03 vs. 0.13 ± 0.03).

Several significant associations were observed between EEG connectivity measures and behavioral or subjective variables. Notably, connectivity in the central region during the walking task under the negative condition was positively correlated with avoidance scores (*rho* = 0.322, *p* = 0.005). Similarly, central connectivity during the elevator task in the negative condition was positively associated with raw and percentage avoidance scores (*rho* = 0.238, *p* = 0.040). A negative correlation was found between frontal connectivity during the neutral walking condition and state anxiety (*rho* = −0.270, *p* = 0.019). Additionally, total simulator sickness scores were negatively correlated with connectivity during the negative walking task (14. Q_neg_walk; *rho* = −0.241, *p* = 0.038).

## 4 Discussion

The primary objective of this study was to investigate the impact of VR environments simulating height on cerebral connectivity during gait. We examined regional connectivity patterns under neutral and negative conditions (simulating height) during two tasks: preparation (elevator task) and active walking (plank walking task). The results revealed distinct connectivity patterns across cortical regions, emphasizing the brain's dynamic adaptations to stress and task demands. Key findings included increased frontal connectivity during the negative condition for both tasks, enhanced central connectivity during gait in the negative condition, and greater occipital connectivity during scene analysis compared to walking. Additionally, network‐level metrics provided further insights into how the brain organizes its activity under varying emotional contexts. Modularity was significantly reduced in the negative condition, suggesting a breakdown in functional segregation when exposed to height‐related stress. Furthermore, eigenvector centrality analyses revealed task‐dependent increases in influence of frontal and parietal nodes, with both regions showing significantly higher centrality during walking than during preparation, indicating their key integrative role in motor control and visuospatial processing under locomotor demands.

A significant finding was the increased connectivity in the frontal region during the negative condition, regardless of the task. This result aligns with prior literature highlighting the prefrontal cortex's critical role in managing stress, emotional regulation, and cognitive control under threat (El Basbasse et al. 2023; Zhu et al. 2023). Rather than assuming conscious motor monitoring, we interpret this frontal recruitment as reflecting a general reconfiguration of network properties in response to emotionally salient stimuli, consistent with findings from functional neuroimaging and EEG studies that associate heightened prefrontal engagement with contextually adaptive processing (Lohmann et al. 2010; Fraschini et al. 2015). This frontal increase, observed during both elevator and walking tasks under the negative condition, may indicate a shift toward integrative processing to support context monitoring and preparatory control mechanisms.

In the central region, greater connectivity was observed, specifically during the gait task under the negative condition. This region, encompassing motor and sensorimotor areas, is pivotal in coordinating motor responses and maintaining stability during locomotion (Schniepp et al. 2014; Zhu et al. 2024). The task‐specific enhancement of central connectivity indicates that dynamic balance demands amplify these regions' recruitment during threatening locomotor tasks. The heightened central connectivity aligns with the concept of “protective” walking behavior (Cleworth et al. 2012; Adkin and Carpenter 2018; Raffegeau, Fawver, Clark, et al. 2020; Raffegeau, Fawver, Young, et al. 2020), characterized by slower gait and increased stance width, aimed at enhancing postural safety in threatening environments. These findings support the notion that under conditions of increased environmental threat, functional networks reorganize to prioritize sensorimotor stability, consistent with models of adaptive motor control (Young et al. 2016; Ellmers et al. 2020). While central connectivity was significantly increased during the walking task under the negative condition, we did not observe significant differences between the elevator and walking tasks per se in the central regions. This lack of a main task effect in motor‐related areas is notable, especially given that active locomotion typically elicits robust sensorimotor engagement. One possible interpretation is that the simulated postural threat in both tasks elicited similar levels of motor system engagement, regardless of actual movement. This could suggest that the perceived risk of instability, rather than the motor act itself, was sufficient to upregulate central connectivity. Alternatively, it may reflect task design constraints, such as limited biomechanical challenge during virtual walking or the stabilizing effect of harness support, which may have dampened task‐related differences. Future studies with greater ecological motor demands or comparisons between overground and virtual walking could help clarify this interpretation.

Occipital connectivity was significantly greater during the preparation task (elevator) compared to walking, independent of the condition, highlighting the pivotal role of visual processing in scene analysis before movement initiation. This finding aligns with evidence that the visual cortex is highly engaged in interpreting spatial layout and environmental cues to inform subsequent motor strategies (Cleworth et al. 2012; Schniepp et al. 2014). Rather than reflecting condition‐specific threat processing, this increased occipital connectivity likely corresponds to task‐related differences in visual demand. During the elevator task, participants remained stationary while observing a virtual scene with vertical motion cues (i.e., the rising elevator), which may have heightened the need for visual tracking and spatial interpretation. In contrast, the walking task involved forward locomotion along a static virtual plank, with different visual flow characteristics. This suggests that the observed occipital activation may be more related to the perceptual and attentional demands of the respective virtual environments than to emotional condition per se. Nonetheless, the enhanced visual processing during preparation underscores the relevance of visual scene evaluation prior to initiating movement, particularly when facing spatial complexity.

Importantly, network‐level metrics provided additional insight into how these connectivity patterns reflect global brain reorganization in response to task and emotional load. We found significantly lower modularity in the negative condition, suggesting decreased functional segregation and a shift toward more integrated network dynamics. This result aligns with findings from Kabbara et al. (2019) and Zippo et al. (2018), who demonstrated that emotional or cognitively demanding tasks promote transient reductions in modularity, reflecting the need for widespread inter‐regional communication. The reduced modularity observed under the height‐induced threat suggests that neural networks reconfigure to support flexible integration of cognitive, emotional, and motor demands.

In contrast, eigenvector centrality revealed task‐related differences: higher centrality values were found during walking in both frontal and parietal regions. This measure reflects a node's influence based on its neighbors' importance (Lohmann et al. 2010; Fraschini et al. 2015), indicating that these regions assume a more central role within the overall network during active locomotion. Such a finding complements the regional connectivity results, showing that these areas also serve as functional hubs integrating distributed information beyond local coupling. Notably, the parietal cortex has been implicated in spatial orientation and movement planning, reinforcing its importance during locomotor tasks under stress.

The results of modularity and eigenvector centrality support a dynamic balance between segregation and integration, depending on task and emotional context. While modularity reduction reflects the dissolution of distinct functional communities under stress, the rise in centrality during gait suggests that certain regions, particularly within the sensorimotor and cognitive control networks, assume enhanced integrative roles. This dynamic interplay aligns with theories of network flexibility and adaptive reconfiguration in response to cognitive and environmental demands (Zippo et al. 2018).

The observed correlations provide additional insights into the functional significance of regional brain connectivity during VR‐based gait under threat. The positive association between central connectivity and avoidance scores in the elevator and walking tasks under the negative condition suggests that individuals who report greater behavioral avoidance tend to exhibit increased sensorimotor network engagement when exposed to height‐related threat. This may reflect a compensatory neural mechanism aimed at maintaining postural control under elevated anxiety or fear responses. Conversely, the negative correlation between frontal connectivity during neutral walking and state anxiety suggests frontal regions' potential protective or regulatory role in modulating affective responses. Higher frontal connectivity may reflect more effective top‐down cognitive control, possibly reducing the subjective experience of anxiety. Furthermore, the negative correlation between simulator sickness and connectivity during the negative walking task indicates that individuals with stronger network integration may better adapt to immersive environments, mitigating adverse physiological symptoms. Together, these findings support the notion that connectivity patterns reflect general task demands and track interindividual variability in affective and physiological responses, aligning with prior work linking functional brain networks to trait‐like behavioral tendencies and situational adaptations (Ellmers et al. 2020). These associations underscore the relevance of considering both neural and behavioral markers when evaluating adaptive motor responses under threat.

While these findings offer valuable insights, several limitations must be acknowledged. The study relied on a relatively homogenous group of healthy young adults, which may limit the generalizability of the results to older adults or individuals with clinical conditions such as acrophobia or motor impairments. Additionally, while EEG connectivity metrics provide valuable information about functional relationships between brain regions, they do not directly capture causal interactions or directional connectivity. Future studies should employ complementary methods, such as dynamic causal modelling or graph theoretical approaches, to further elucidate these interactions. Despite these limitations, the findings have important clinical implications. By identifying specific neural adaptations during locomotion in anxiety‐inducing environments, this research can inform the development of VR‐based therapies for anxiety disorders and balance impairments, offering targeted interventions that leverage the brain's natural adaptability to challenging conditions.

## Author Contributions

**Layla Cupertino:** conceptualization, software, formal analysis, writing – original draft. **Emanuele Los Angeles:** conceptualization, software, formal analysis, writing – original draft. **Nathalia Mendes Pellegrino:** software, formal analysis, writing – original draft. **Thayna Magalhães‐Novaes:** validation, formal analysis, visualization. **Brenda Luciano de Souza:** validation, data curation, visualization. **Mohamed Bouri:** writing – review and editing. **Daniel Boari Coelho:** conceptualization, software, formal analysis, supervision, writing – review and editing.

## Conflicts of Interest

The authors declare no conflicts of interest.

### Peer Review

The peer review history for this article is available at https://www.webofscience.com/api/gateway/wos/peer‐review/10.1111/ejn.70131.

## Supporting information


**Data S1** Supporting information

## Data Availability

The EEG dataset analyzed in this study is available from the corresponding author upon reasonable request. All scripts and analysis routines used for EEG preprocessing, connectivity computation, and graph‐theoretical analyses are provided as Supporting Information.

## References

[ejn70131-bib-0001] Adkin, A. L. , and M. G. Carpenter . 2018. “New Insights on Emotional Contributions to Human Postural Control.” Frontiers in Neurology 9: 789. 30298048 10.3389/fneur.2018.00789 PMC6160553

[ejn70131-bib-0002] Baker, B. L. , D. C. Cohen , and J. T. Saunders . 1973. “Self‐Directed Desensitization for Acrophobia.” Behaviour Research and Therapy 11: 79–89. 4781961 10.1016/0005-7967(73)90071-5

[ejn70131-bib-0003] Bassett, D. S. , and O. Sporns . 2017. “Network Neuroscience.” Nature Neuroscience 20: 353–364. 28230844 10.1038/nn.4502 PMC5485642

[ejn70131-bib-0004] Beck, A. T. 1961. “An Inventory for Measuring Depression.” Archives of General Psychiatry 4: 561. 13688369 10.1001/archpsyc.1961.01710120031004

[ejn70131-bib-0005] Bullmore, E. , and O. Sporns . 2009. “Complex Brain Networks: Graph Theoretical Analysis of Structural and Functional Systems.” Nature Reviews. Neuroscience 10: 186–198. 19190637 10.1038/nrn2575

[ejn70131-bib-0006] Cleworth, T. W. , B. C. Horslen , and M. G. Carpenter . 2012. “Influence of Real and Virtual Heights on Standing Balance.” Gait & Posture 36: 172–176. 22464634 10.1016/j.gaitpost.2012.02.010

[ejn70131-bib-0007] Diemer, J. , N. Lohkamp , A. Mühlberger , and P. Zwanzger . 2016. “Fear and Physiological Arousal During a Virtual Height Challenge—Effects in Patients With Acrophobia and Healthy Controls.” Journal of Anxiety Disorders 37: 30–39. 26600469 10.1016/j.janxdis.2015.10.007

[ejn70131-bib-0008] El Basbasse, Y. , J. Packheiser , J. Peterburs , et al. 2023. “Walk the Plank! Using Mobile Electroencephalography to Investigate Emotional Lateralization of Immersive Fear in Virtual Reality.” Royal Society Open Science 10: 221239. 37266038 10.1098/rsos.221239 PMC10230188

[ejn70131-bib-0009] Ellmers, T. J. , A. J. Cocks , E. C. Kal , and W. R. Young . 2020. “Conscious Movement Processing, Fall‐Related Anxiety, and the Visuomotor Control of Locomotion in Older Adults.” Journals of Gerontology: Series B 75: 1911–1920. 10.1093/geronb/gbaa081 PMC7566972 32761087

[ejn70131-bib-0010] Fraschini, M. , A. Hillebrand , M. Demuru , L. Didaci , and G. L. Marcialis . 2015. “An EEG‐Based Biometric System Using Eigenvector Centrality in Resting State Brain Networks.” IEEE Signal Processing Letters 22: 666–670.

[ejn70131-bib-0011] Gage, W. H. , R. J. Sleik , M. A. Polych , N. C. McKenzie , and L. A. Brown . 2003. “The Allocation of Attention During Locomotion Is Altered by Anxiety.” Experimental Brain Research 150: 385–394. 12707746 10.1007/s00221-003-1468-7

[ejn70131-bib-0012] Kabbara, A. , M. Khalil , G. O'Neill , et al. 2019. “Detecting Modular Brain States in Rest and Task.” Network Neuroscience 3: 878–901. 31410384 10.1162/netn_a_00090 PMC6663471

[ejn70131-bib-0013] Krijn, M. , P. M. G. Emmelkamp , R. Biemond , C. De Wilde De Ligny , M. J. Schuemie , and C. A. P. G. Van Der Mast . 2004. “Treatment of Acrophobia in Virtual Reality: The Role of Immersion and Presence.” Behaviour Research and Therapy 42: 229–239. 14975783 10.1016/S0005-7967(03)00139-6

[ejn70131-bib-0014] Lohmann, G. , D. S. Margulies , A. Horstmann , et al. 2010. “Eigenvector Centrality Mapping for Analyzing Connectivity Patterns in fMRI Data of the Human Brain.” PLoS ONE 5: e10232. 20436911 10.1371/journal.pone.0010232 PMC2860504

[ejn70131-bib-0015] Meehan, M. , B. Insko , M. Whitton , and F. P. Brooks . 2002. “Physiological Measures of Presence in Stressful Virtual Environments.” ACM Transactions on Graphics 21: 645–652.

[ejn70131-bib-0016] Peterson, S. M. , E. Furuichi , and D. P. Ferris . 2018. “Effects of Virtual Reality High Heights Exposure During Beam‐Walking on Physiological Stress and Cognitive Loading.” PLoS ONE 13: e0200306. 29979750 10.1371/journal.pone.0200306 PMC6034883

[ejn70131-bib-0017] Raffegeau, T. E. , S. A. Brinkerhoff , M. Clark , et al. 2024. “Walking (and Talking) the Plank: Dual‐Task Performance Costs in a Virtual Balance‐Threatening Environment.” Experimental Brain Research 242: 1237–1250. 38536454 10.1007/s00221-024-06807-w PMC11078829

[ejn70131-bib-0018] Raffegeau, T. E. , B. Fawver , M. Clark , et al. 2020. “The Feasibility of Using Virtual Reality to Induce Mobility‐Related Anxiety During Turning.” Gait & Posture 77: 6–13. 31951915 10.1016/j.gaitpost.2020.01.006

[ejn70131-bib-0019] Raffegeau, T. E. , B. Fawver , W. R. Young , A. M. Williams , K. R. Lohse , and P. C. Fino . 2020. “The Direction of Postural Threat Alters Balance Control When Standing at Virtual Elevation.” Experimental Brain Research 238: 2653–2663. 32944785 10.1007/s00221-020-05917-5 PMC8364805

[ejn70131-bib-0020] Schniepp, R. , G. Kugler , M. Wuehr , et al. 2014. “Quantification of Gait Changes in Subjects With Visual Height Intolerance When Exposed to Heights.” Frontiers in Human Neuroscience 8: 963. 10.3389/fnhum.2014.00963. 25538595 PMC4255593

[ejn70131-bib-0021] Starcke, K. , and M. Brand . 2012. “Decision Making Under Stress: A Selective Review.” Neuroscience & Biobehavioral Reviews 36: 1228–1248. 22342781 10.1016/j.neubiorev.2012.02.003

[ejn70131-bib-0022] Stergiadis, C. , V.‐D. Kostaridou , and M. A. Klados . 2022. “Which BSS Method Separates Better the EEG Signals? A Comparison of Five Different Algorithms.” Biomedical Signal Processing and Control 72: 103292.

[ejn70131-bib-0023] Teng, C. , L. Cong , Q. Tian , et al. 2024. “EEG Microstate in People With Different Degrees of Fear of Heights During Virtual High‐Altitude Exposure.” Brain Research Bulletin 218: 111112. 39486463 10.1016/j.brainresbull.2024.111112

[ejn70131-bib-0024] Wuehr, M. , K. Breitkopf , J. Decker , G. Ibarra , D. Huppert , and T. Brandt . 2019. “Fear of Heights in Virtual Reality Saturates 20 to 40 m Above Ground.” Journal of Neurology 266: 80–87. 31102019 10.1007/s00415-019-09370-5

[ejn70131-bib-0025] Young, W. R. , M. Olonilua , R. S. W. Masters , S. Dimitriadis , and A. Mark Williams . 2016. “Examining Links Between Anxiety, Reinvestment and Walking When Talking by Older Adults During Adaptive Gait.” Experimental Brain Research 234: 161–172. 26403296 10.1007/s00221-015-4445-z PMC4713710

[ejn70131-bib-0026] Zhu, H. Y. , H.‐T. Chen , and C.‐T. Lin . 2023. “The Effects of Virtual and Physical Elevation on Physiological Stress During Virtual Reality Height Exposure.” IEEE Transactions on Visualization and Computer Graphics 29: 1937–1950. 34898434 10.1109/TVCG.2021.3134412

[ejn70131-bib-0027] Zhu, H. Y. , H.‐T. Chen , and C.‐T. Lin . 2024. “Understanding the Effects of Stress on the P300 Response During Naturalistic Simulation of Heights Exposure.” PLoS ONE 19: e0301052. 38630669 10.1371/journal.pone.0301052 PMC11023450

[ejn70131-bib-0028] Zippo, A. G. , P. A. Della Rosa , I. Castiglioni , and G. E. M. Biella . 2018. “Alternating Dynamics of Segregation and Integration in Human EEG Functional Networks During Working‐Memory Task.” Neuroscience 371: 191–206. 29246785 10.1016/j.neuroscience.2017.12.004

